# The Pathology and Diagnosis of Diseases of the Chest, Comprising a Rational Exposition of Their Signs; with an Appendix Containing Various Opinions and Experiments on the Motions and Sounds of the Heart and on the Bronchi

**Published:** 1841-01

**Authors:** 


### PART SECOND.

### Bibliographical Notices.

---

Art. I.—*The Pathology and Diagnosis of Diseases of the Chest, comprising a Rational Exposition of their Signs; with an Appendix, containing various opinions and experiments on the Motions and Sounds of the Heart and on the Bronchi.* By Charles J. B. Williams, m.d. f.r.s., Professor of Medicine in University College, &c. *Fourth Edition, much enlarged and illustrated by Plates and Tables.—London,* 1840. 8vo, pp. 331.

The number of editions this book has gone through being sufficiently indicative of its merits, we might be content to let it pass unnoticed were it not for the particular importance of the subject of which it treats, and the admirable manner in which this is handled by the author. We are sorry to observe that the practice of auscultation, notwithstanding the many excellent teachers of it in the metropolis, is still very imperfectly studied in our schools, and, consequently, that a large proportion of the junior members of the profession enter into practice incapacitated for the most effective treatment of a large proportion of the diseases they meet with. It is for this reason we have always been anxious to employ our pages in promoting the cause of the physical diagnosis of chest-diseases; and we now feel it no less a duty than a pleasure to recommend to the notice of our younger readers the manual of Dr. Williams now before us. It is, without question, the very best of the kind that exists in any language; and it is so full of well-digested and well-arranged knowledge, that there are few practised auscultators who will not benefit by its perusal; to the student and young auscultator it is indispensable.

The work is divided into three parts. The first contains two chapters, one devoted to the Physical Examination of the Chest, the other to the Examination of the Chest through the vital properties or functions of its organs: the second part treats of the Pathology and Physical Signs of the Diseases of the Bronchi, Pleura, and Lung: and the third is devoted to the Pathology and Diagnosis of Diseases of the Heart and Great Vessels. This last part is divided into five chapters, treating, respectively, of the physical examination of the heart; of functional diseases of the heart; of inflammatory diseases of the heart; of structural diseases of the heart; of diseases of the great vessels. The Appendix contains several Reports, formerly published, on the Motions and Sounds of the Heart, and a new "Report (by the Author) of Experiments on the Contractility and Sensibility of the Lungs and Air-tubes," read at the meeting of the British Association in 1840.

We regret extremely that the press of other matter prevents us from making any further extracts from this volume than the brief summary given by the author of the results of his experiments on the contractility of the lungs and air-tubes, contained in the last memoir. The experiments themselves are very interesting, and the enquiry one of much practical importance.

"I trust that many results of the preceding experiments are sufficiently evident without much further comment. Almost all of them prove that the air-tubes are possessed of irritable contractility, excitable by electric, chemical, and mechanical stimuli. The contractibility resembles that of the intestines, or of the arteries more than that of voluntary muscles or of the œsophagus, the contractions and relaxations being gradual, not sudden. They are, however, much less tardy than those of the arteries. The irritability of the contractile bronchial fibres is speedily exhausted by continued stimulation, and may be in some degree restored by rest, even in the lung removed from the body for an hour or more. The contractility of these fibres seems much influenced, in case of sudden death, by the mode of death, being for a short time suspended after death caused by a blow on the back of the neck, and sometimes by death caused by pithing, not by hemorrhage. Several vegetable poisons destroy or impair this contractility. Extracts of stramonium and belladonna produced this effect most completely. Strychnia, extract of conium, and bimeconate morphia, also, to a great degree. Hydrocyanic acid, on the other hand, did not impair it at all. The action of these poisons on the bronchial fibres does not correspond with that on other contractile tissue, such as the heart and arteries, œsophagus and intestines, and the voluntary muscles; these in many cases having retained their irritability when that of the bronchi had been destroyed. It is possible that some medicinal agents (strychnia for example) may impair the contractility of the bronchial fibres by fixing them in a state of tonic spasm. The preceding experiments do not determine this point. The different state of the trachea, in some contracted, so that the ends of the cartilaginous rings overlap; in others expanded, so that they do not form three fourths of the caliber of the tube, seems to countenance this supposition, and deserves further investigation. The bronchial fibres seem not to be excitable through the nerves of the lungs; for mechanical irritation of these nerves produced no effect on them, and passing the electric current through the nerves to the lungs was much less effectual in causing their contractions than when the current was passed through the trachea." (pp. 330-1.)

